# Virtual reconstruction of midfacial bone defect based on generative adversarial network

**DOI:** 10.1186/s13005-022-00325-2

**Published:** 2022-06-27

**Authors:** Yu-Tao Xiong, Wei Zeng, Lei Xu, Ji-Xiang Guo, Chang Liu, Jun-Tian Chen, Xin-Ya Du, Wei Tang

**Affiliations:** 1 State Key Laboratory of Oral Diseases and National Clinical Research Centre for Oral Diseases and Department of Oral and Maxillofacial Surgery, West China Hospital of Stomatology, Sichuan University, No.14, 3rd section of Ren Min Nan Road, Chengdu, 610041 China; 2 Machine Intelligence Laboratory, College of Computer Science, Sichuan University, Chengdu, 610065 China; 3 Department of Stomatology, the People’s Hospital of Longhua, Shenzhen, 518109 China

**Keywords:** Virtual Surgical Planning, Midface Reconstruction, Generative Adversarial Networks

## Abstract

**Background:**

The study aims to evaluate the accuracy of the generative adversarial networks (GAN) for reconstructing bony midfacial defects.

**Methods:**

According to anatomy, the bony midface was divided into five subunit structural regions and artificial defects are manually created on the corresponding CT images. GAN is trained to reconstruct artificial defects to their previous normal shape and tested. The clinical defects are reconstructed by the trained GAN, where the midspan defects were used for qualitative evaluation and the unilateral defects were used for quantitative evaluation. The cosine similarity and the mean error are used to evaluate the accuracy of reconstruction. The Mann–Whitney U test is used to detect whether reconstruction errors were consistent in artificial and unilateral clinical defects.

**Results:**

This study included 518 normal CT data, with 415 in training set and 103 in testing set, and 17 real patient data, with 2 midspan defects and 15 unilateral defects. Reconstruction of midspan clinical defects assessed by experts is acceptable. The cosine similarity in the reconstruction of artificial defects and unilateral clinical defects is 0.97 ± 0.01 and 0.96 ± 0.01, *P* = 0.695. The mean error in the reconstruction of artificial defects and unilateral clinical defects is 0.59 ± 0.31 mm and 0.48 ± 0.08 mm, *P* = 0.09.

**Conclusion:**

GAN-based virtual reconstruction technology has reached a high accuracy in testing set, and statistical tests suggest that it can achieve similar results in real patient data. This study has preliminarily solved the problem of bony midfacial defect without reference.

**Supplementary Information:**

The online version contains supplementary material available at 10.1186/s13005-022-00325-2.

## Background

The bony midface has a complex structure and is very important for facial appearance and function [1, 2]. Various reasons may cause midfacial defects [3, 4], and how to restore the normal shape and function of the midface has become an urgent clinical problem [5, 6]. Imaging data, such as CT, CBCT, MRI, etc. can be used to reconstruct the soft and hard tissue morphology [7, 8]. However, due to the lack of normal imaging data as a reference, doctors can only rely on personal experience for surgery [4, 9–11].

**Fig. 1:**
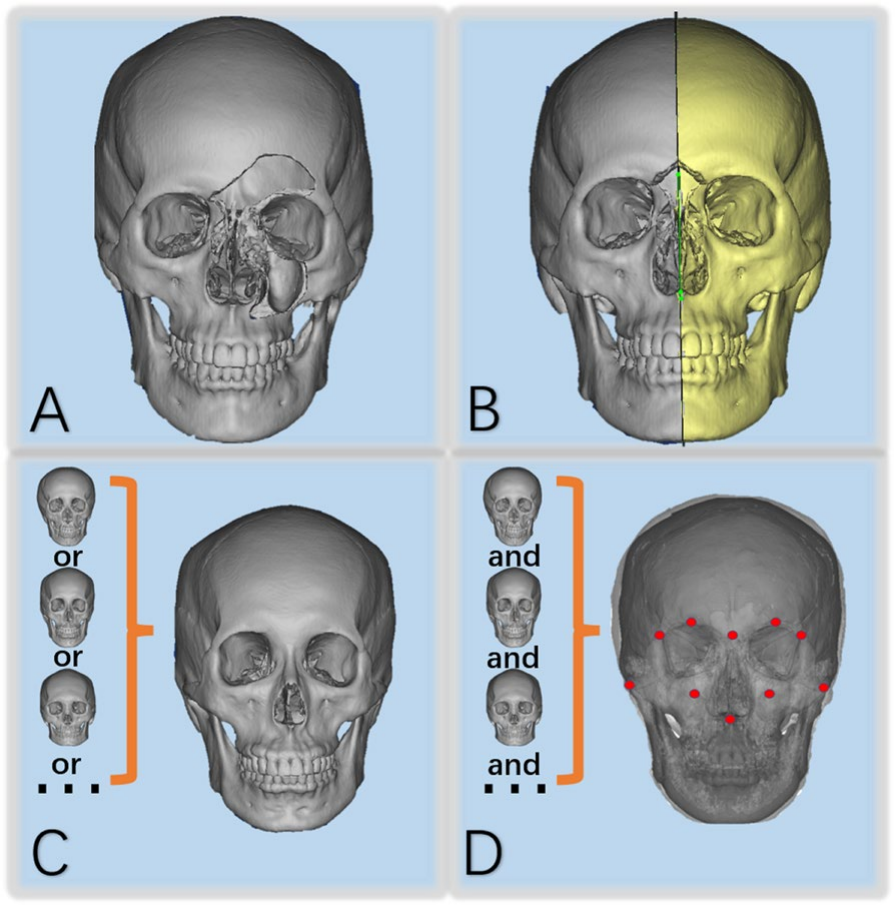
Main reconstruction methods of facial bone defects: **A** Three-dimensional image of a facial bone defect. **B** The mirror technology. **C** The normal template technology. **D** The deformable template technology

Virtual reconstruction methods of facial bone mainly include mirror technology, normal template and deformable template technology [2, 12–15] (Fig. 1). The mirror technology is the most classic, and its basic principle is to project the normal skull of the healthy side onto the affected side to repair the defect [7, 16–18]. However, it cannot use for midspan and bilateral defects. Although the normal template and the deformable template technology fill the gap in this part, the repeatability and interactivity restrict their clinical application [13, 15, 19]. Therefore, there is an urgent need for a method to achieve intelligent reconstruction of bony midface in clinical practice.

Ideal virtual bone reconstruction should base on the remaining bone information to obtain more accurate additional information to repair the defect accurately. The deep learning technology makes the above process become reality, which could learn from a large number of complex samples to find specific rules by imitating the human brain [6, 20, 21]. As a new deep learning algorithm, GAN is widely used in the field of medical data processing, showing excellent image generation ability [22–24].

## Methods

This study aims to use GAN to repair the bony midfacial defect and evaluate its accuracy. To address the research purpose, CT data of the healthy person and real patient were selected from the database of digital cloud platform (http://dsurgery.cn/huaxicloud/) which is a craniofacial CT database of West China Hospital of Stomatology and a display platform for virtual surgery plans. The inclusion criteria of the former were as follows: 1) having no obvious facial deformity 0.2) the interlayer spacing of CT images was 1 mm with tube voltage 120 kV and tube current 335 mA. The inclusion criteria of the latter was that the defects must be confined to bony midface.

This study was approved by the Ethics Committee of West China Hospital of Stomatology, and the safe use of health and medical data was also ensured.

### Data acquisition

The normal and real patient images of CT data in DICOM format were downloaded from the database of digital cloud platform.

### Artificial defects construction

**Fig. 2:**
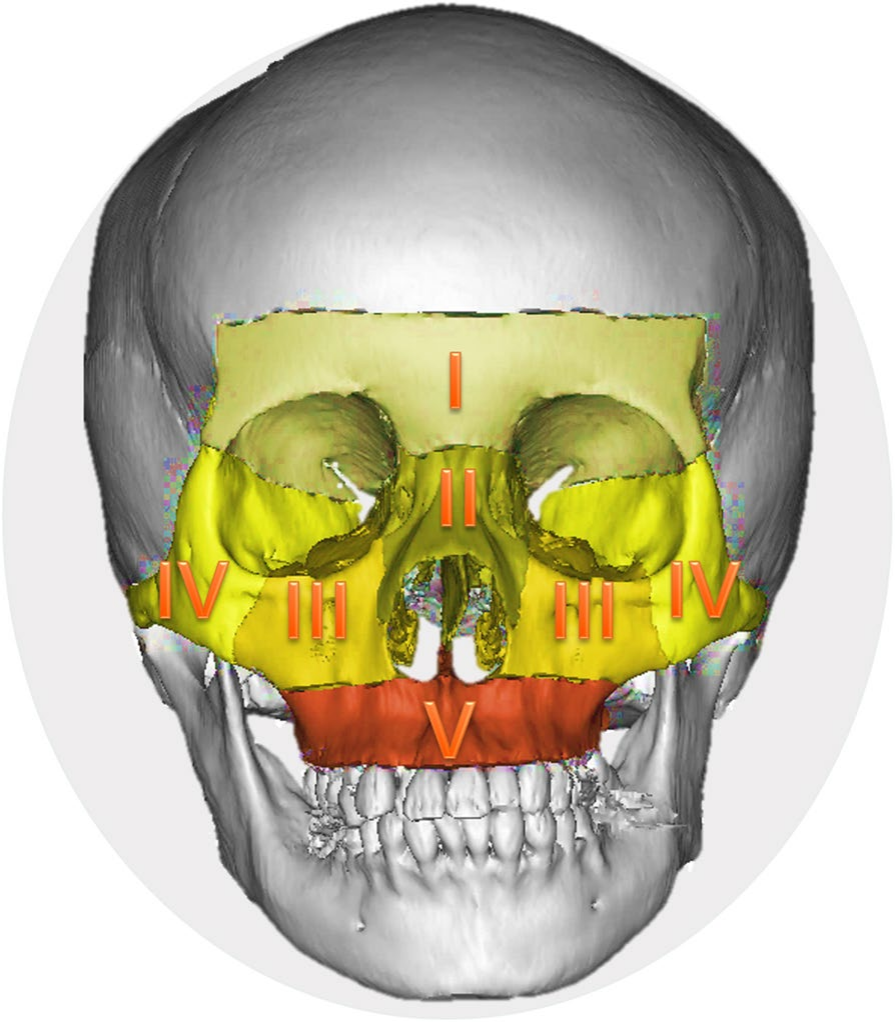
Sub-unit partition of midfacial bone: frontal sinus-frontal bone (**I**), naso-orbital-ethmoid (**II**), infraorbital margin-maxillary sinus (**III**), zygomatic complex (**IV**), and residual maxilla (**V**)

**Fig. 3:**
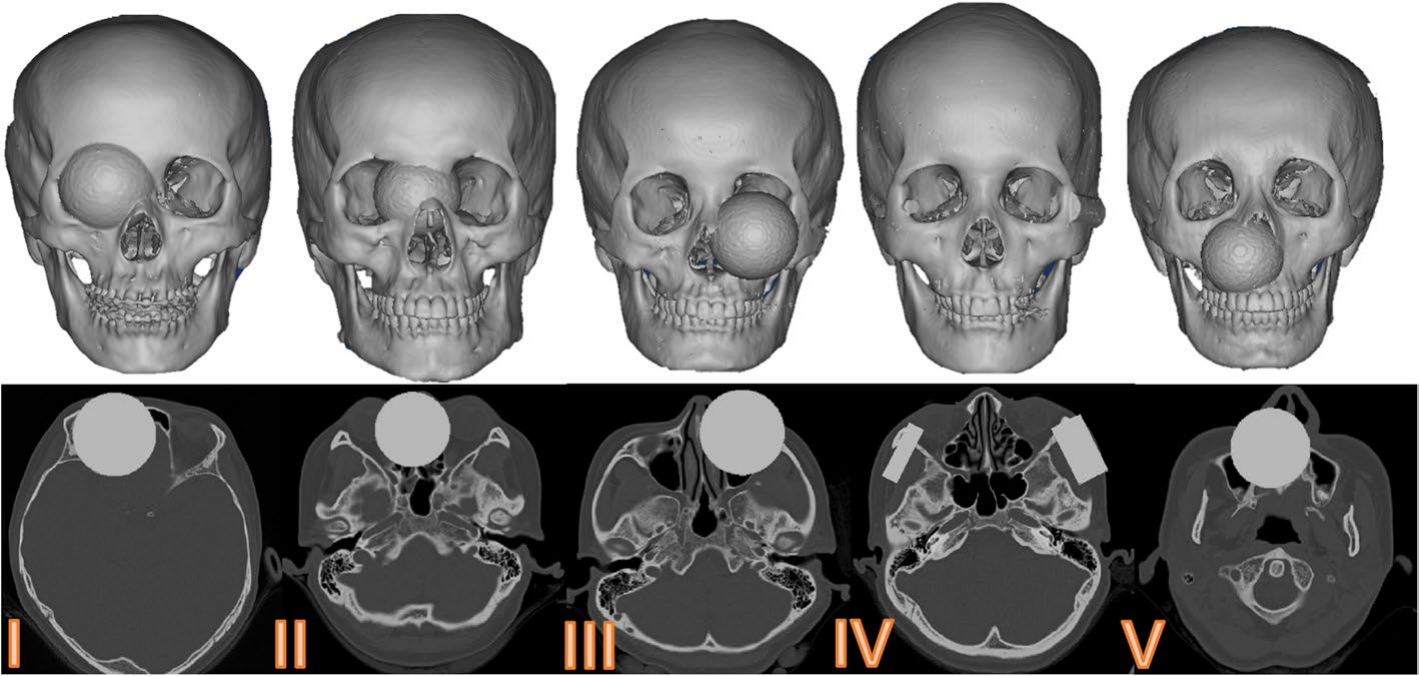
Five kinds of artificial defect construction of bony midface

According to the anatomy, the bony midface was divided into five sub-unit structural regions (Fig. 2). Normal CT data were imported into Mimics 16.0 (Materialise, Leuven, Belgium) to obtain the normal 3D model. Then sphere, cuboid or semi-cylindrical phantoms were constructed in the model to simulate bone defects (Fig. 3). Finally, the artificial defect images were obtained and exported in DICOM format for later use.

### GAN completes training and testing

**Fig. 4:**
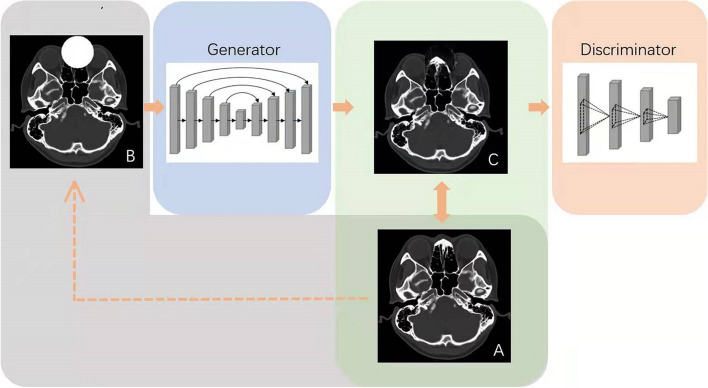
Basic structure of GAN: The normal image (**A**) is artificially constructed to form a defect image (**B**), and the generator generates a reconstructed image (**C**). Discriminator verifies the similarity between normal image (**A**) and reconstructed image (**C**)

The normal data and corresponding artificial defects were imported into GAN for training. All data are divided into training set and testing set with a ratio of 8:2. The GAN structure used in this study is shown in Fig. 4, which contains a generator and a discriminator. Its interference factors were artificially set as follows to improve learning difficulty and prevent overfitting: 1) Image preprocessing-including random horizontal flipping of CT slices (probability 0.5); 2) random scaling with ratio from 0.8 to 1.2; 3) random rotation (maximum rotation angle was 11°); 4) adding Gaussian noise.

The generative model is a Resnet101 [25] pretrained on ImageNet, which takes the random variable z and the defect image x with resolution of 512 × 512 as the inputs, and the output is the reconstructed image G(x,z) with resolution of 512 × 512. The discriminative model takes x and the generated G(x,z) as inputs, followed by 3 convolution layers with kernel size of 3, batch normalization with leakyReLU [26] as activation function, and outputs a comprehensive similarity score D(x,G(x,z)). The model is implemented via PyTorch [27] which is already publicly available and Adam [28] optimizer with the initial learning rate of 0.002. The server is Linux OS with the hardware of CPU Intel Xeon E5-2620 @2.4 GHz, four NVIDIA Tesla Titan GPUs, and 128 GB of RAM. After GAN completes training, the reconstructed images in JPG format were generated. These images can be imported into separate software for evaluation.

### Clinical defects reconstruction

**Fig. 5:**
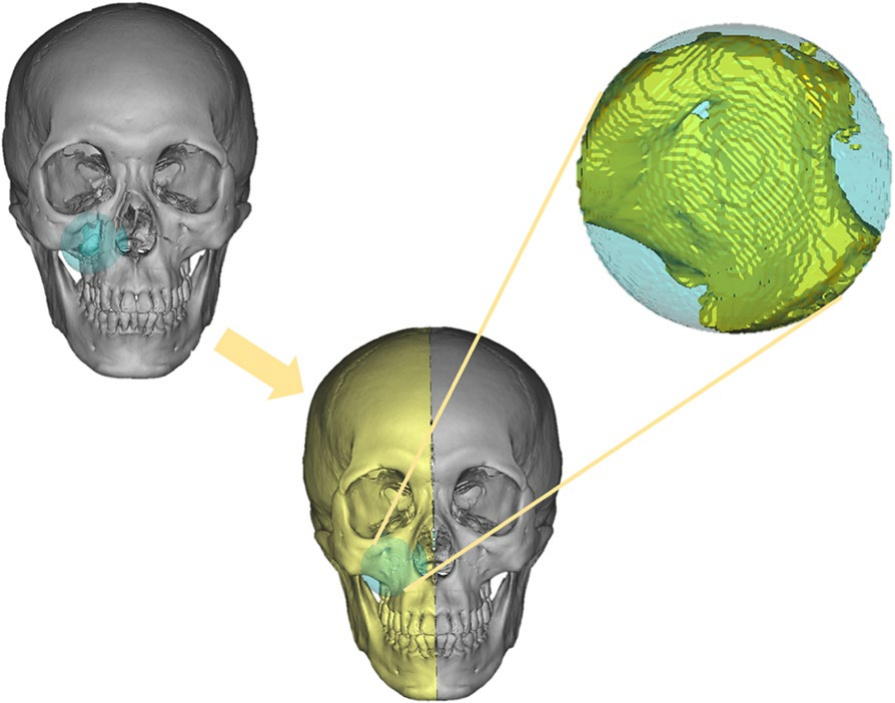
Delineation of the region of interest and usage of mirror technology

The real patient data were imported into Mimics 16.0 to obtain the clinical 3D model. A surgeon placed an aforesaid phantom on the model to cover the defect, and imported the model into GAN for reconstructing. For unilateral defects, the surgeon additionally used mirror technology to reconstruct the same delineated area. Specifically, a series of anatomic landmarks on the facial midsagittal plane were selected to represent the mirroring plane [29]. Combined with fine manual adjustment, the reconstructed shape of a mirror technology can be considered correct (Fig. 5).

### Accuracy assessment

Firstly, the reconstructed images were paired with the correct images to calculate the cosine similarity, which is a significant indicator to evaluate the similarity of two objects [30]. The CT images are converted to grayscale images with 8 bits per pixel using a Python script (Python version 3.9). Then, the reconstructed images were imported into MIMICS 16.0 to obtain the reconstructed 3D model. The reconstructed 3D model and the correct 3D model were imported into Geomagic Studio 2013 software (Raindrop Geomagic Studio 2013®, Raindrop Geomagic, Inc., NC, USA) for registering and calculating the mean error of the two surfaces. As for midspan clinical defects, 4 experienced maxillofacial/plastic surgeons were invited to evaluate the effect of the reconstructed area. The content of the evaluation is anatomical similarity, edge continuity, and whether the overall shape meets physiological and aesthetic requirements.

### Statistical analysis

The SPSS 26.0 statistical software package (IBM, Armonk, NY, USA) was used for data analysis. All values are presented as the mean ± standard deviation. For the two-group comparison, *P* values were derived from the Mann–Whitney U test to determine differences between groups with unbalanced non-normal data. For all comparisons, *P* < 0.05 was considered statistically significant.

## Results

**Fig. 6:**
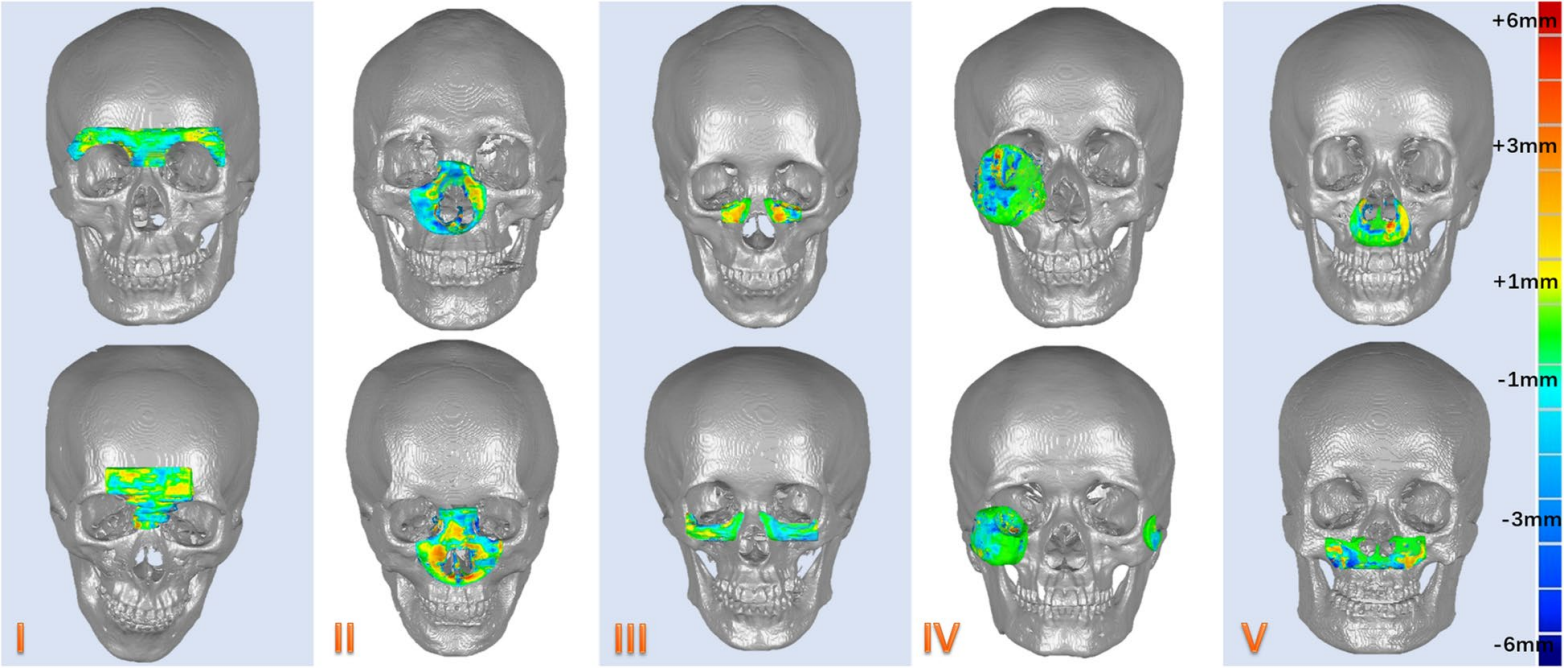
Partial reconstruction effect of five sub-units in the artificial defects

**Fig. 7:**
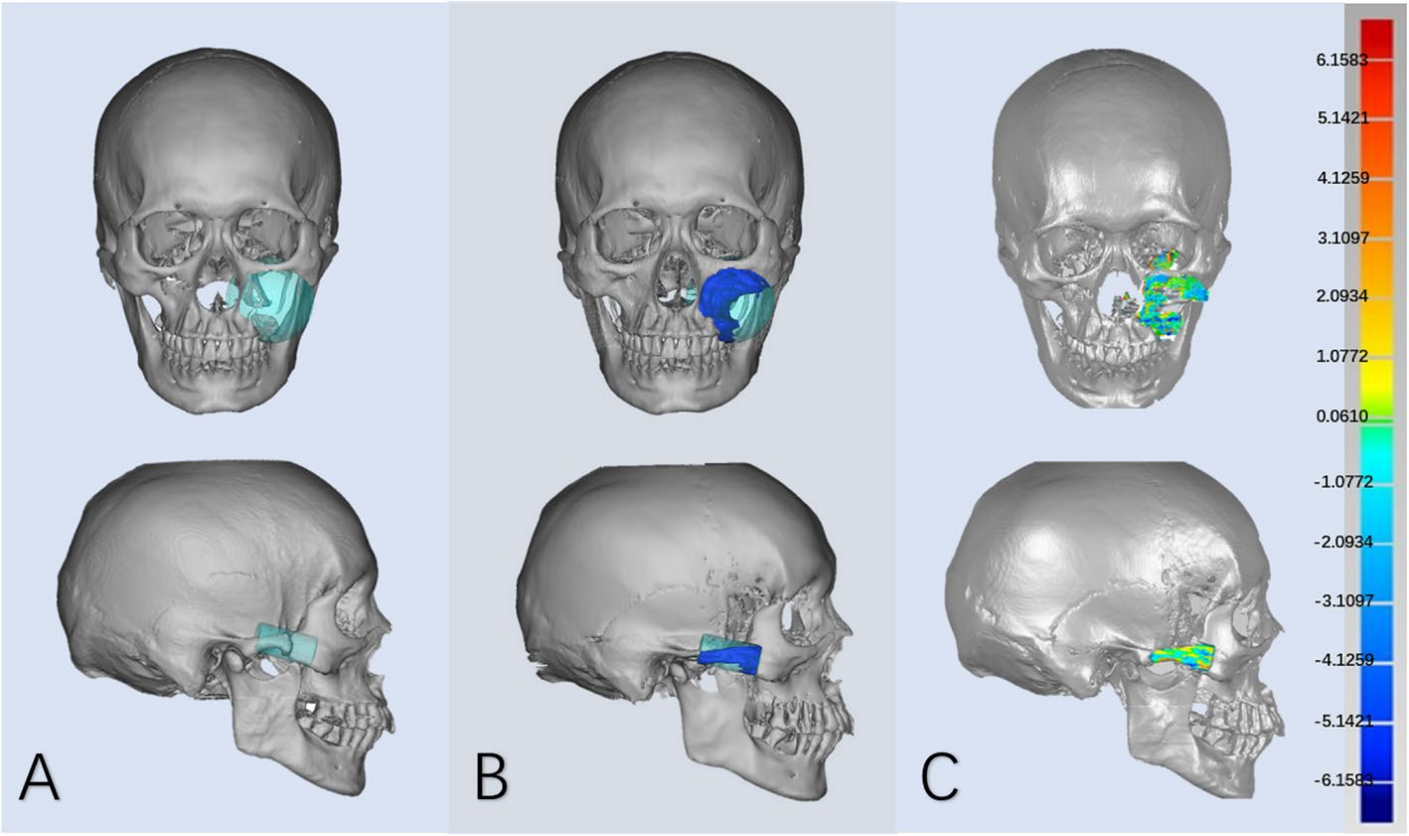
Partial reconstruction effect in the unilateral clinical defects: **A** Three-dimensional model of clinical defect. **B** Reconstructed model **C** Registration of the reconstructed model with the correct reference

This study included 518 normal CT data, with 415 in training set and 103 in testing set, and 17 real patient data, of which 15 cases of unilateral defects were included in the statistical analysis. The model was trained for 40 h on Linux OS with the hardware of CPU Intel Xeon E5-2620 @2.4 GHz, four NVIDIA Tesla Titan GPUs, and 128 GB of RAM. The cosine similarity and the mean error in the reconstruction of artificial defects are respectively 0.97 ± 0.01 and 0.59 ± 0.31 mm. The sub-units and the overall situation are shown in Table 1. The reconstruction effect of partial artificial defect is shown in Fig. 6. The cosine similarity and the mean error in the reconstruction of clinical defects are respectively 0.96 ± 0.01 and 0.48 ± 0.08 mm. Causes of defect and accuracy of reconstruction in 15 cases of real patient data are shown in Table 2. The reconstruction effect of partial unilateral clinical defect is shown in Fig. 7.

**Table 1:** The results of the cosine similarity and the mean error of the reconstruction of artificial defects in each sub-unit and overall

region	cosine similarity	mean error (mm)
I	0.97 ± 0.01	0.39 ± 0.22
II	0.98 ± 0.01	0.78 ± 0.27
III	0.96 ± 0.01	0.64 ± 0.30
IV	0.96 ± 0.01	0.44 ± 0.27
V	0.97 ± 0.01	0.65 ± 0.32
**overall**	**0.97 ± 0.01**	**0.59 ± 0.31**

**Table 2:** The causes of defect and the results of the cosine similarity and the mean error of the reconstruction of clinical defects in each case

case	causes of defect	cosine similarity	mean error (mm)
#1	deformity	0.96	0.43
#2	tumor	0.94	0.61
#3	tumor	0.95	0.45
#4	fracture	0.97	0.44
#5	deformity	0.98	0.41
#6	deformity	0.98	0.34
#7	fracture	0.96	0.42
#8	fracture	0.96	0.44
#9	deformity	0.98	0.44
#10	deformity	0.96	0.46
#11	tumor	0.97	0.44
#12	tumor	0.96	0.44
#13	tumor	0.95	0.63
#14	tumor	0.96	0.56
#15	tumor	0.94	0.62
**average**	**-**	**0.96**	**0.48**

**Fig. 8:**
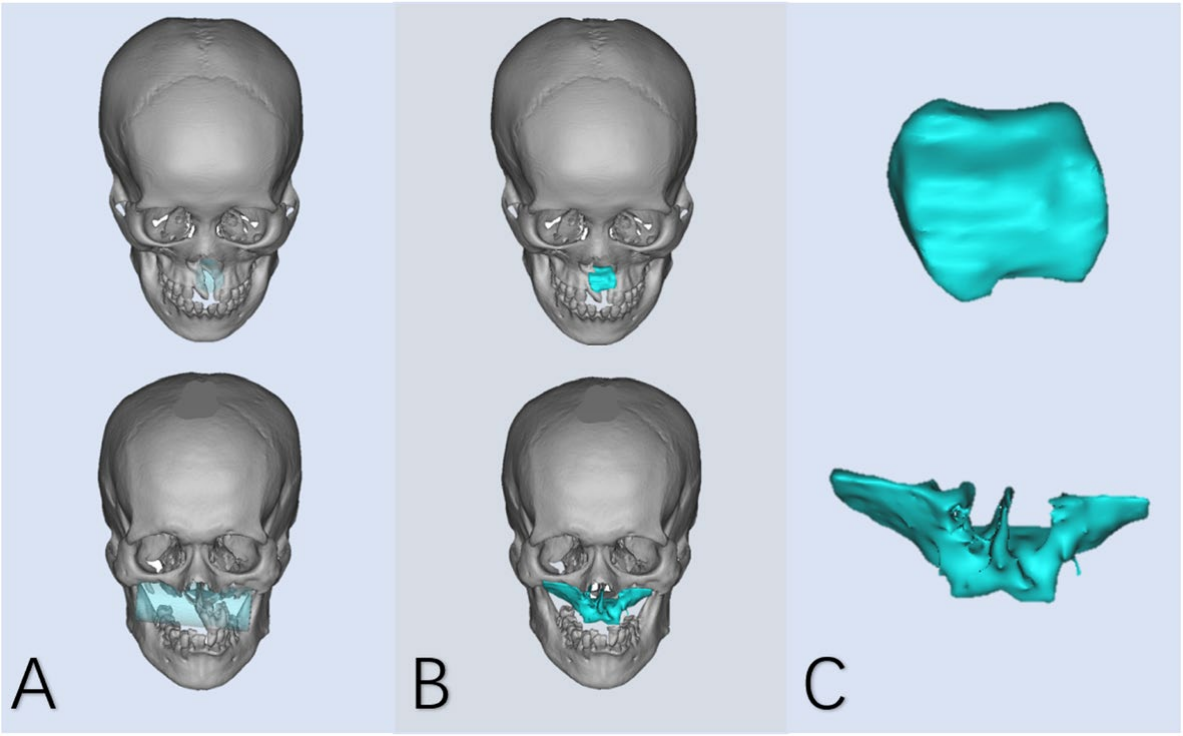
Reconstruction effect in the midspan clinical defects: **A** Three-dimensional model of clinical defect and region of interest. **B** Reconstructed model. **C** The shape of the reconstructed bone block

Figure 8 shows 3D models and reconstructed models of two midspan clinical defects, which was considered acceptable by experts.

**Fig. 9:**
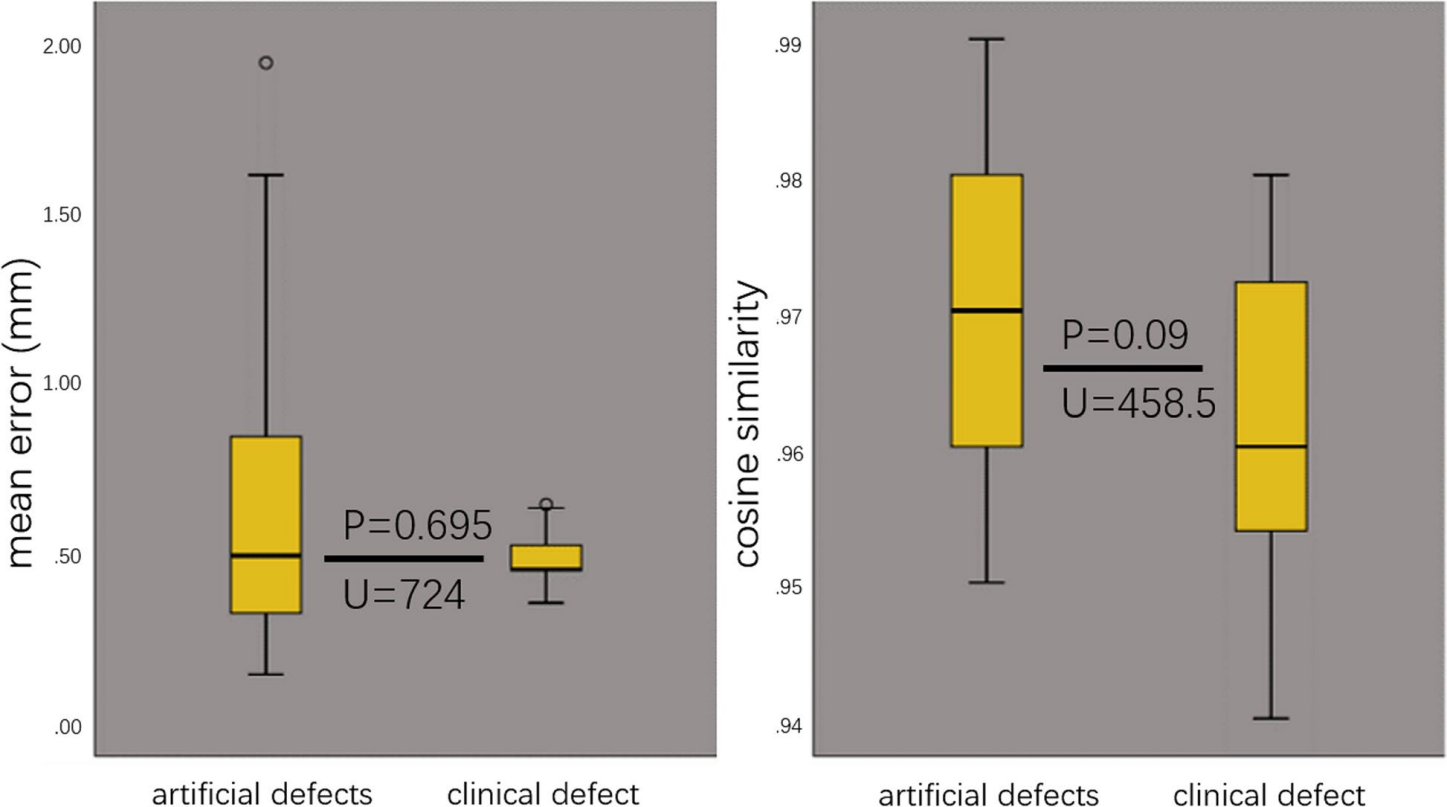
Comparison of the cosine similarity and the mean error (mm) for reconstruction of artificial defects and unilateral clinical defect

The box plots show that the reconstruction accuracy for unilateral clinical defects and artificial defects is similar in distribution (Fig. 9). The results show that the difference between the cosine similarity (*P* = 0.695 > 0.05, U = 724) and the mean error (*P* = 0.09 > 0.05, U = 459) in the two types of defects’ reconstruction were not statistically significant.

## Discussion

The virtual reconstruction technology provides a crucial reference for the reconstruction of the defect in the bony midface, which is a contribution to priori operative procedure [31, 32]. Fuessinger et al. exploited statistical shape model (SSM) to reconstruct naso-orbital-ethmoid and infraorbital region (similar to region II and III in this study), with the mean error of 0.81 mm and 0.75 mm respectively [9]. GASS et al. improved SSM with an average error of 0.26–0.34 mm in orbital floor reconstruction (part of region III) [13]. Semper-Hogg et al. also used SSM to reconstruct zygomatic complex defects (similar to region IV), with a mean error of 0.85 mm [33]. Combining the SSM and Convolution neural network (CNN) which is used to obtain the initial bone shape obtained from pre-traumatic photographs, Xiao et al. reconstructed the large facial defects with a mean error of 3.68 mm [34]. Comparing to the reconstruction results above, the mean error of 0.78 mm in region II, 0.64 mm in region III、and 0.44 mm in region IV were obtained, which indicates that the GAN technology has a higher accuracy. However, it is worth noting that region II has the highest mean error among the five sub-units, which may be due to the more complex cavity structure in the naso-orbital-ethmoid region. These error values can only be used as a reference and other error evaluation methods need to be involved.

It is worth criticizing that most studies did not consider the accuracy of the reconstruction of clinical data or only gave a subjective evaluation [35]. However, real patient data often lack the correct reference. Kodym et al. took the expert-designed cranial implant as the ideal shape of reconstruction [35]. This study used a similar method, through mirror technology to get the correct reference. Through manual fine adjustment, the reconstructed bone block could fit the defect region well. Due to limited clinical data, we used the Mann–Whitney U test to prove whether the good performance in artificial defect reconstruction can be applied to the clinic. The statistical results had shown that the reconstruction effect of GAN-based virtual reconstruction technology on normal data and real patient data is the same.

The main principle of GAN-based virtual reconstruction technology is to reconstruct and repair these defect images layer by layer. Therefore, this study uses cosine similarity to evaluate the similarity between reconstructed images and normal images. The principle is to judge whether the direction of the vector is consistent by transforming the picture into a high-dimensional vector and calculating the cosine value of the angle formed by the two vectors. After the image is represented by vector, the cosine similarity between the reconstructed image $\mathop{a}\limits^{\rightharpoonup}$ and the corresponding normal image $\mathop{b}\limits^{\rightharpoonup}$ is calculated. Therefore, the closer cosine similarity is 1, the more accurate it is.

Experts believe that the reconstruction effect of the midspan defects is acceptable, where mirroring technology cannot be applied in this situation. This article found that the effect of reconstruction is not as “excellent” as that of artificial defects. Overfitting of the training set, complexity of clinical cases, and possible artefacts can all affect the accuracy of the reconstruction. But this is enough to save a lot of work, leaving the surgeon with some optional finishing.

Previous virtual reconstruction methods have their own shortcomings. Mirror technology, the gold standard, can only be applied to the unilateral bone defect. The asymmetry of the human craniomaxillofacial bone itself also brings errors to the mirroring technique [36]. The normal template technology looks for normal human skull as a reference [12]. However, it is very difficult to match craniomaxillofacial bones with complex three-dimensional structures only by the naked eye or linear parameters. The manual operation of our method is just to select the reconstruction range of CT images. The total training process takes 40 h, and the reconstruction of a single case only takes 7–8 min, which makes GAN technology more efficient than the above technologies. But there is a high demand for equipment to build GAN networks, so the software of GAN-based technology and open network platform are our next goal.

Based on the GAN technology and preoperative virtual design software, this study optimized the procedure of preoperative virtual plan, which directly draws the region of interest. Taking the reconstruction results as a reference, the new preoperative virtual plan can also be applied to tumor reconstruction, fracture reduction and contour shaping. The quick and simple strategy can realize automatic, intelligent and personalized design of virtual reconstruction, which is more suitable for clinical application. Through the application on artificial and clinical defect, we find the GAN-assisted virtual reconstruction has a higher accuracy, stability and feasibility compared the above methods.

Taking normal human data as a reference for defect repair has become the mainstream of virtual repair, which heavily relies on the quality of data. Many observational reports have shown that gender and age may affect the parameters of the three-dimensional morphology of craniofacial bones [37, 38]. Including population of different genders and ages as much as possible could balance the samples. The generalization and accuracy of the GAN technology could be improved through the following two ways: The one is to expand the number of samples, rationalizing the composition of training set, validation set and test set data to prevent overfitting; The other is to establish the identification system in the database, in order to improve the sample quality. By screening CT data with similar characteristics to the target to be reconstructed, better results can be achieved on the basis of small sample training. The method can also be applied to other bones or other digital imaging techniques.

## Conclusion

The GAN-based virtual reconstruction technology can achieve accurate reconstruction. Compared with existing technologies, GAN-based technology has higher accuracy and has the potential to become the standard technology.

## Supplementary Information

**Additional file 1.** (PDF 624 kb)

**Additional file 2.** (PDF 873 kb)

## Data Availability

The data presented in this study are available on request from the corresponding author.

## Abbreviations

GAN: Generative adversarial network
CT: Computerized tomography
CBCT: Cone-beam computed tomography
MRI: Magnetic resonance imaging
DICOM: Digital Imaging and Communications in Medicine
SSM: Statistical shape model

## Declarations

### Footnotes

This article has two supplements. Additional file 1 shows the CT image reconstructed by GAN, and Additional file 2 shows 3D models of unilateral clinical data from 15 cases.
