# Supplementary material for: Virtual reconstruction of midfacial bone defect based on generative adversarial network
Source: Head Face Med. 2022 Jun 27;18:19. doi: 10.1186/s13005-022-00325-2 (PMC9235085; doi:10.1186/s13005-022-00325-2)

CT images reconstructed in the test set. (Choose one case for each subunit)

32(Number in normal data set)

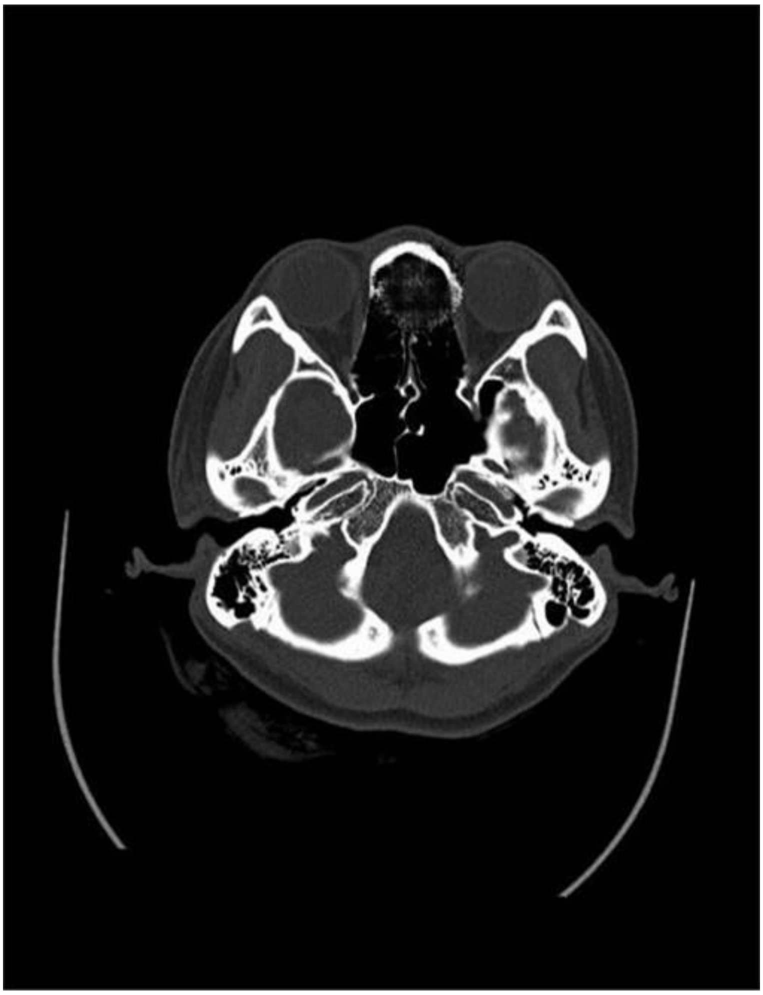

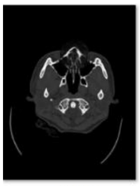
102.jpg

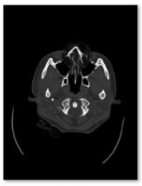
103.jpg

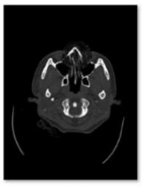
104.jpg

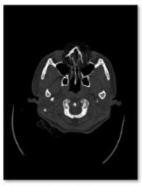
105.jpg

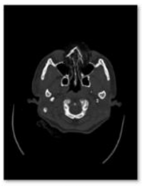
106.jpg

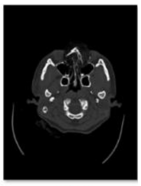
107.jpg

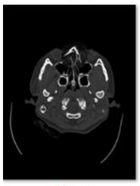
108.jpg

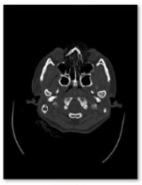
109.jpg

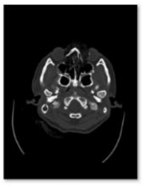
110.jpg

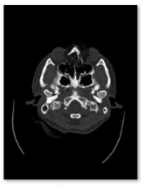
111.jpg

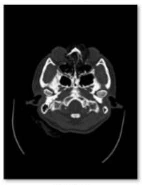
112.jpg

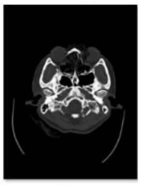
113.jpg

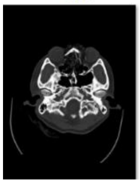

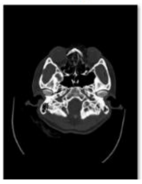

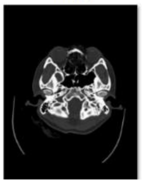

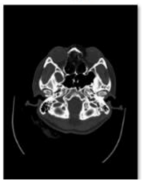

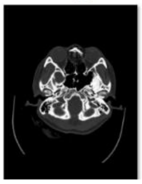

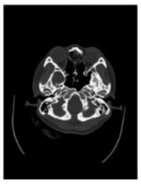

130(Number in normal data set) II

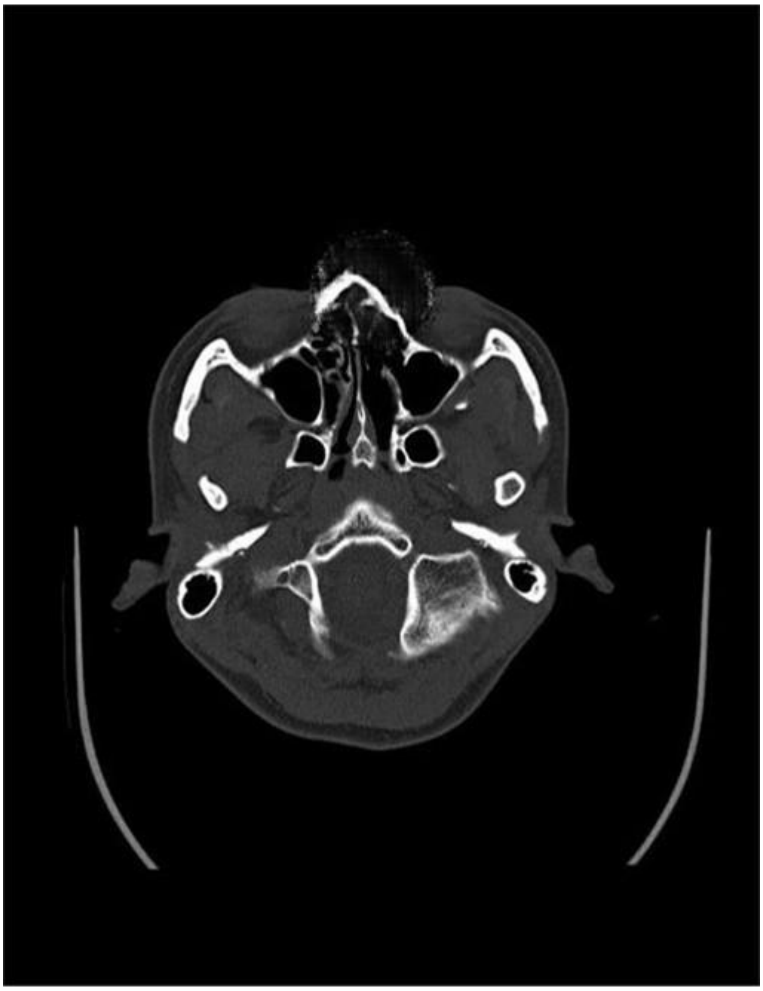

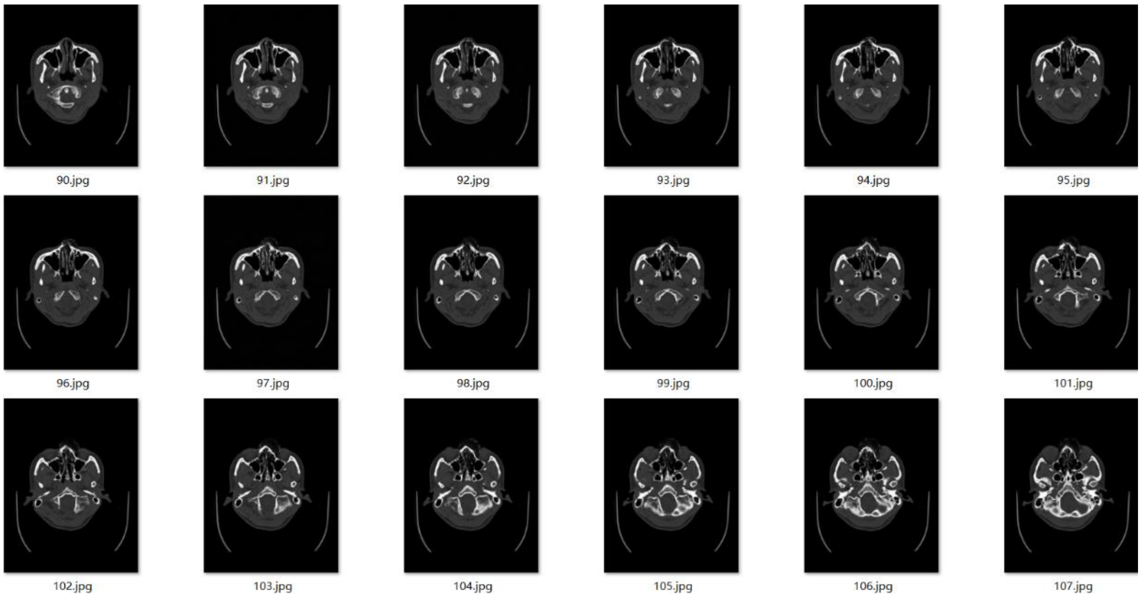

64(Number in normal data set) III

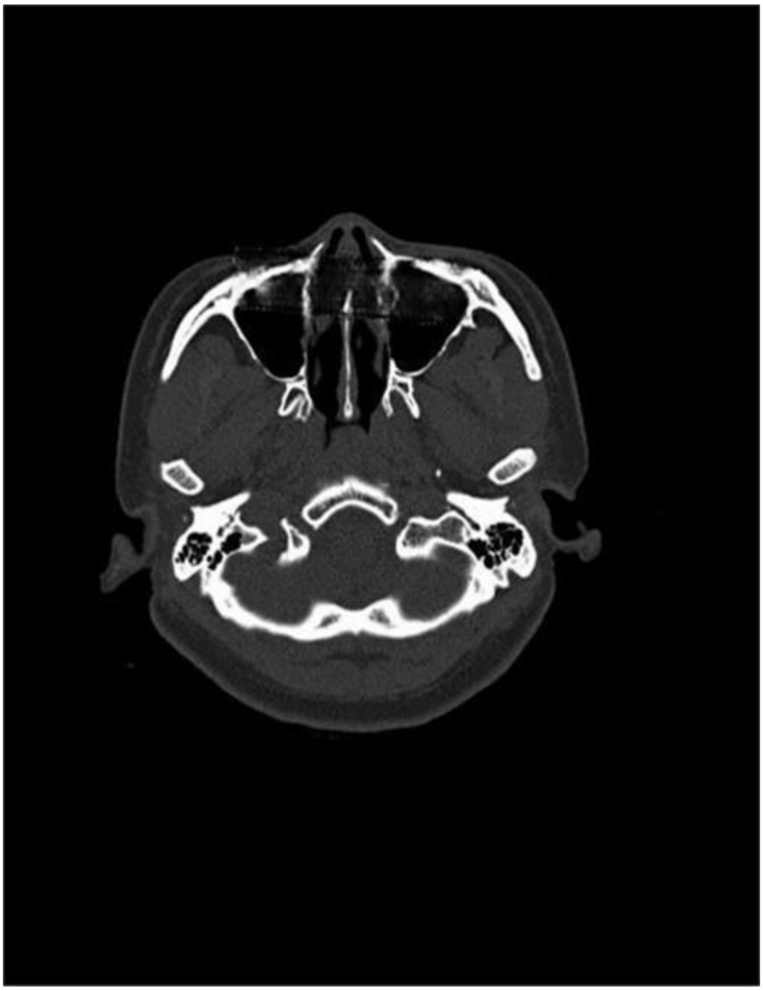

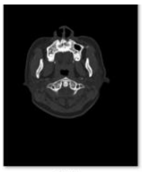

66.jpg

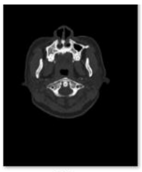

67.jpg

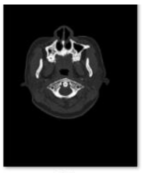

68.jpg

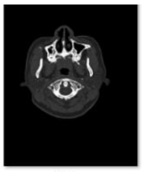

69.jpg

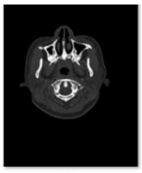

70.jpg

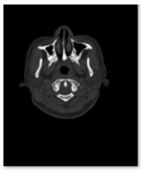

71.jpg

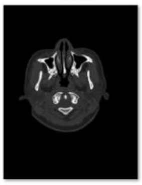

72.jpg

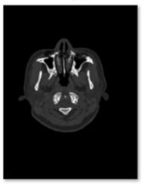

73.jpg

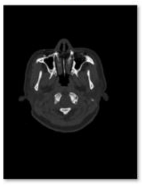

74.jpg

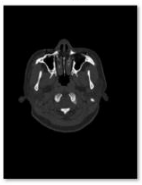

75.jpg

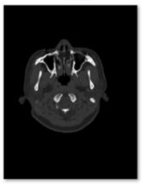

76.jpg

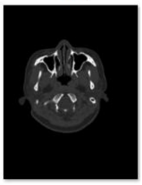

77.jpg

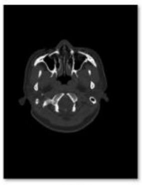

78.jpg

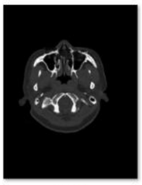

79.jpg

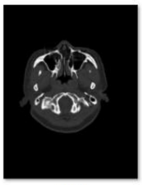

80.jpg

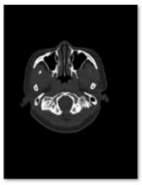

81.jpg

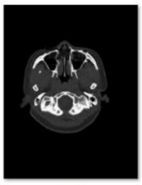

82.jpg

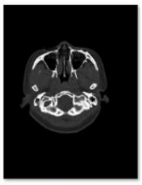

83.jpg

102(Number in normal data set) Ⅳ

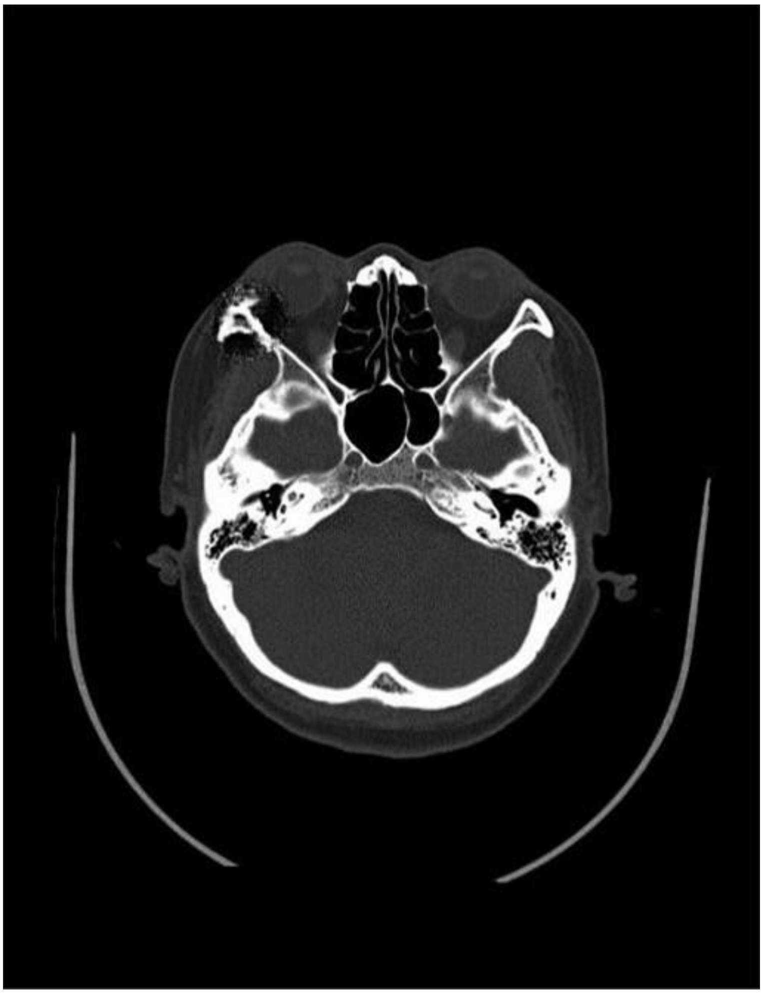

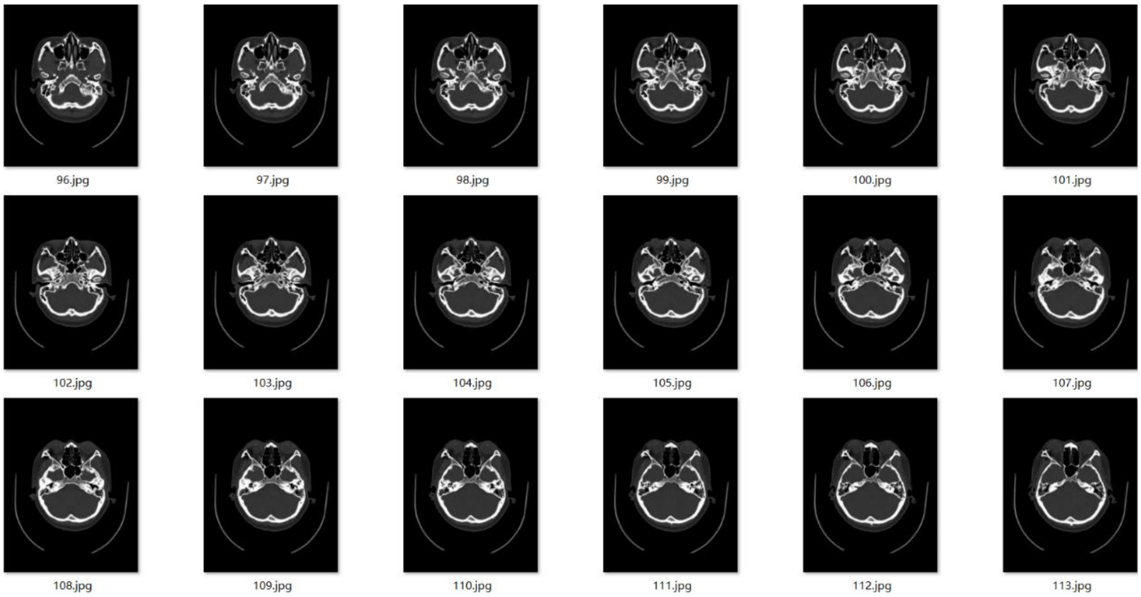

110(Number in normal data set) V

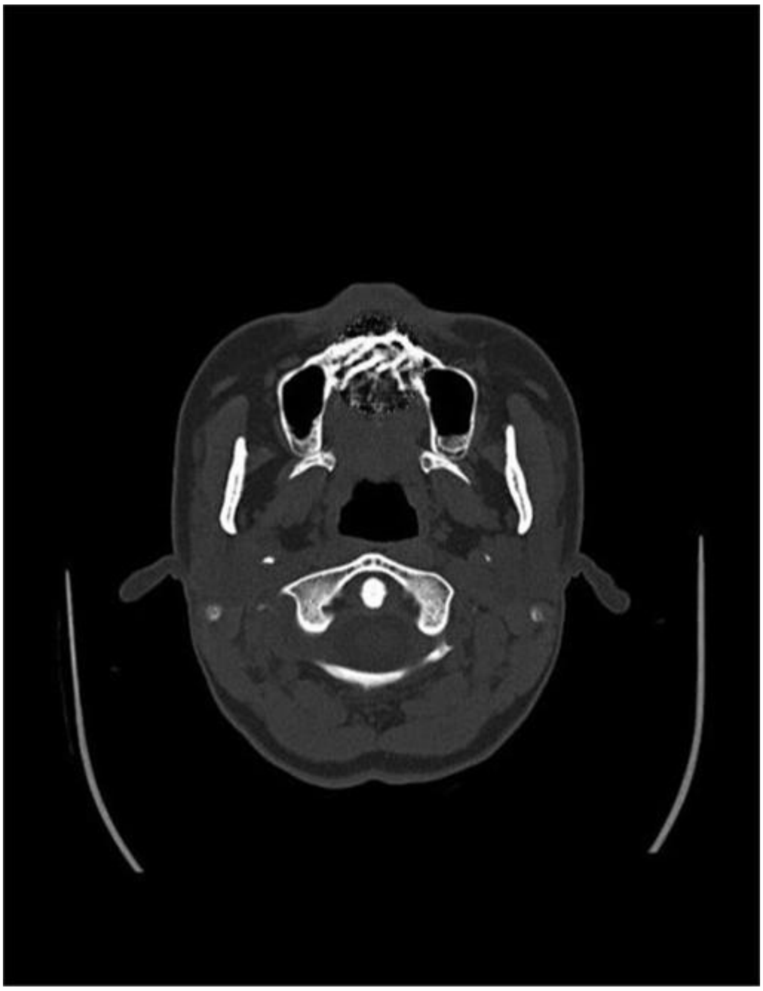

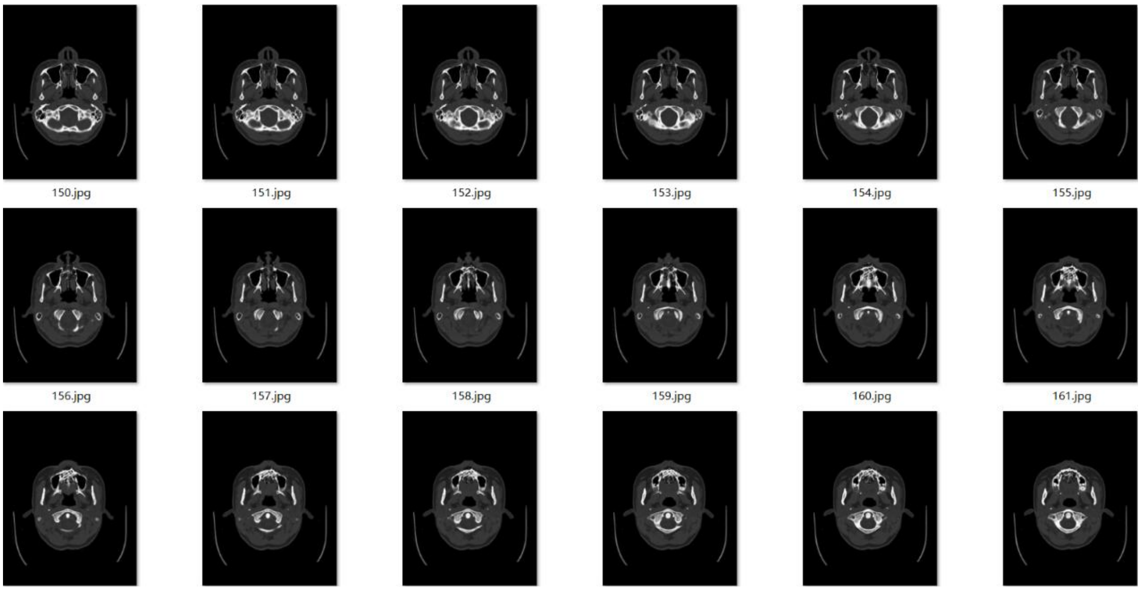

Supplement: Supplementary file 1 — Additional file 1. (PDF 624 kb) [file 13005_2022_325_MOESM1_ESM.pdf]
