# Supplementary material for: Virtual reconstruction of midfacial bone defect based on generative adversarial network
Source: Head Face Med. 2022 Jun 27;18:19. doi: 10.1186/s13005-022-00325-2 (PMC9235085; doi:10.1186/s13005-022-00325-2)
Supplement: Supplementary file 2 — Additional file 2. (PDF 873 kb) [file 13005_2022_325_MOESM2_ESM.pdf]

Three-dimensional reconstruction of 15 cases of clinical defects and delineation of regions of interest (based on Mimics 16.0)

#1	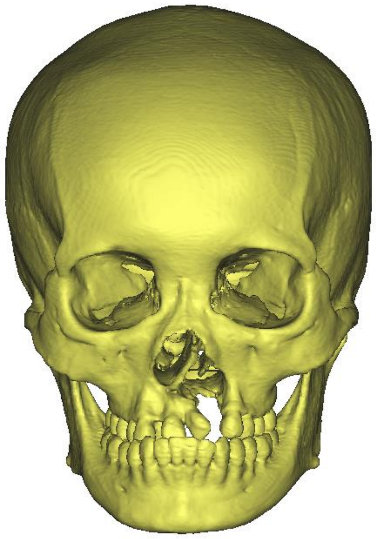	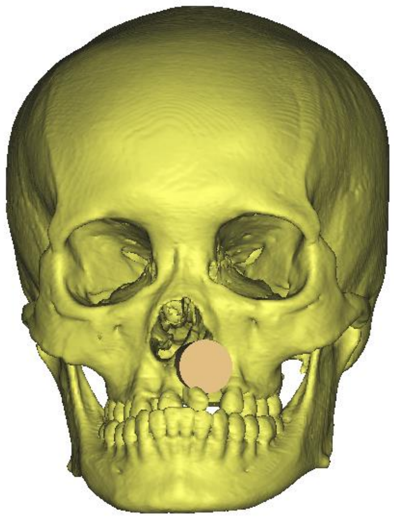
#2	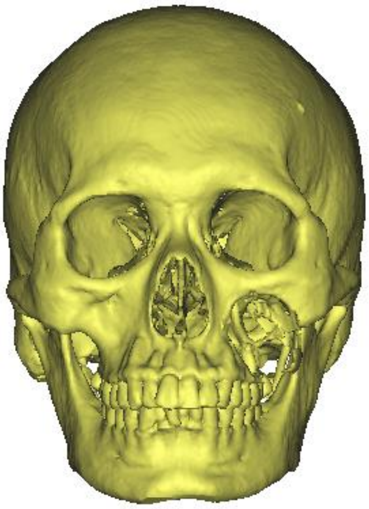	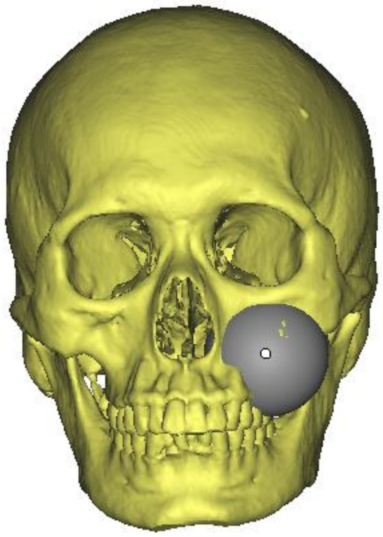

#3	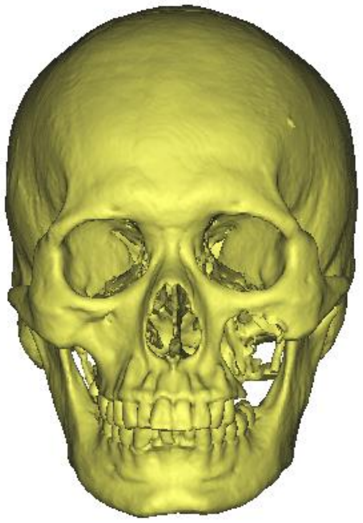	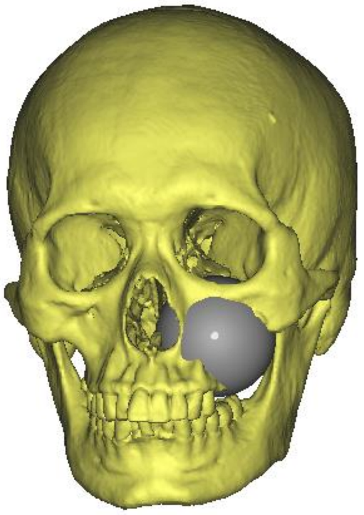
#4	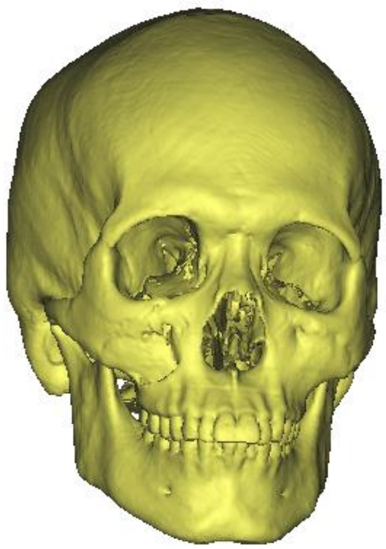	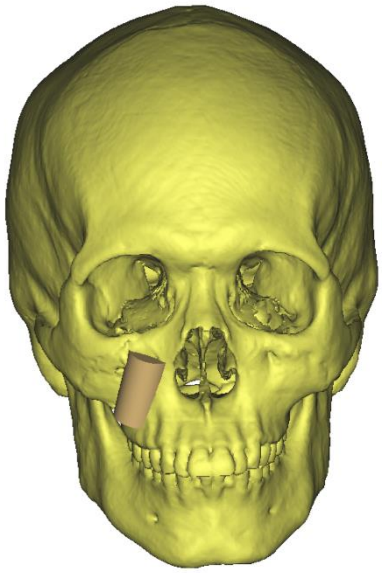

#5	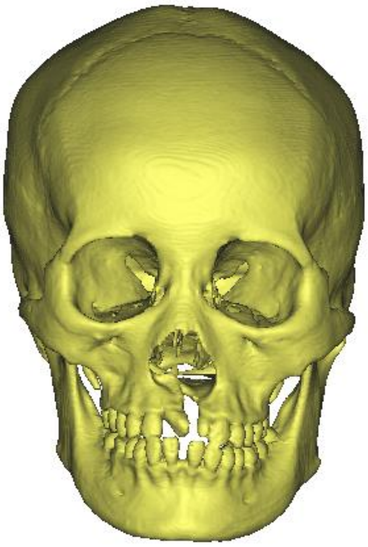	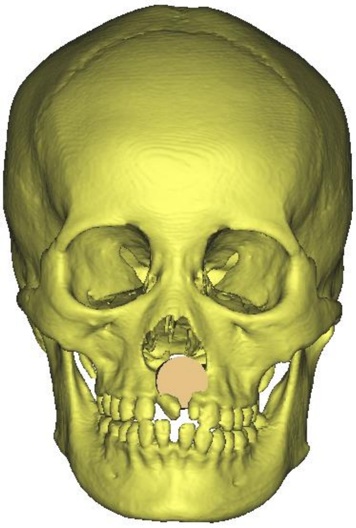
#6	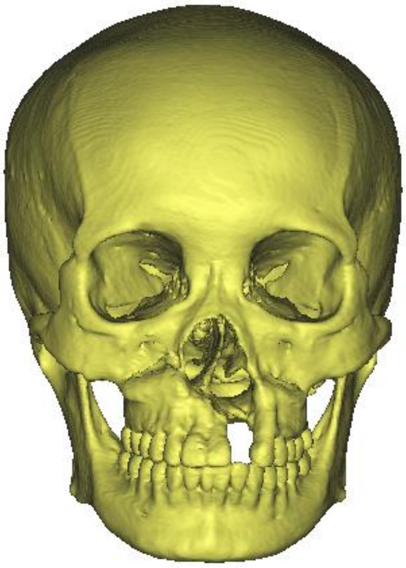	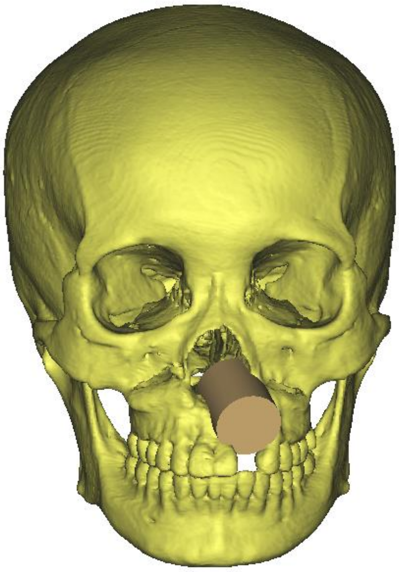

#7	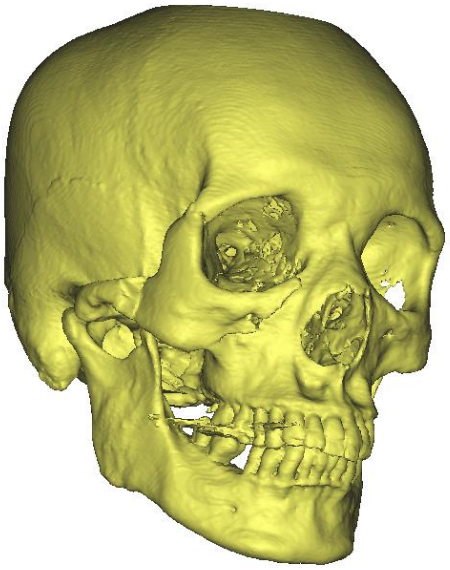	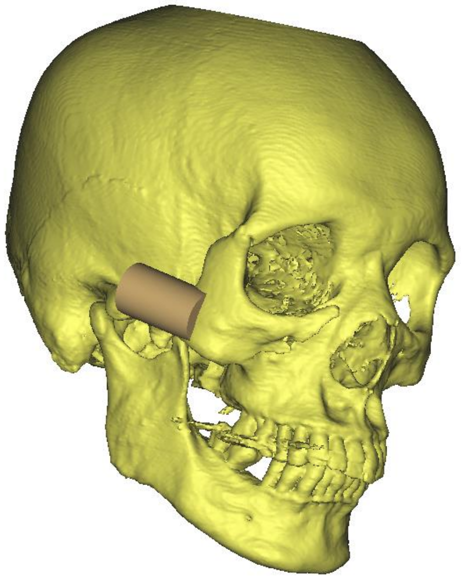
#8	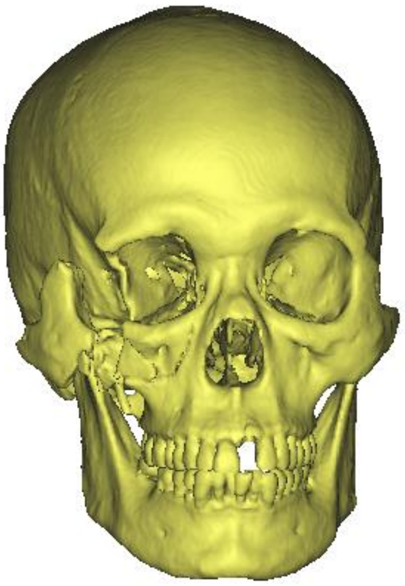	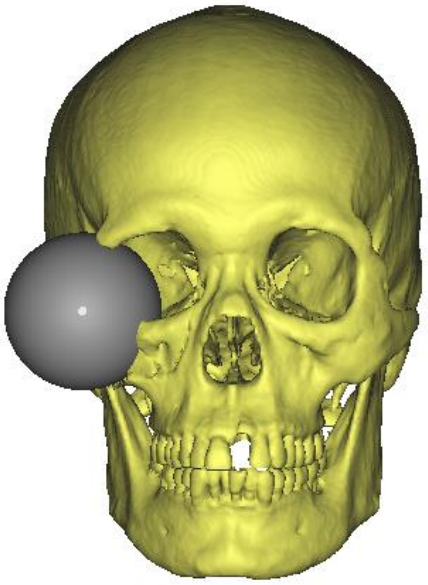

#9	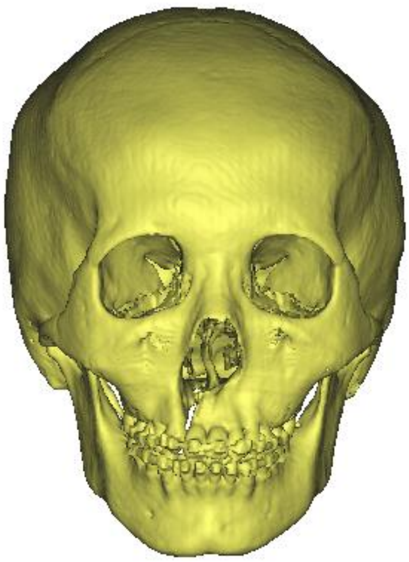	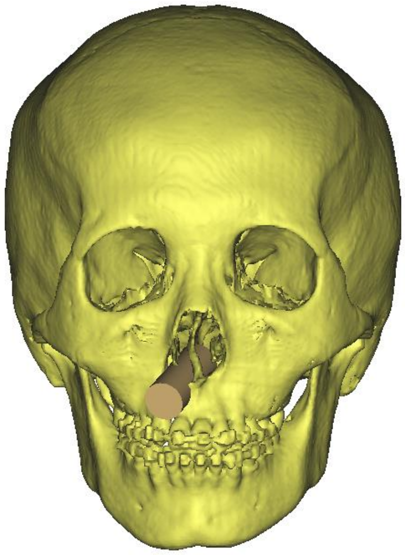
#10	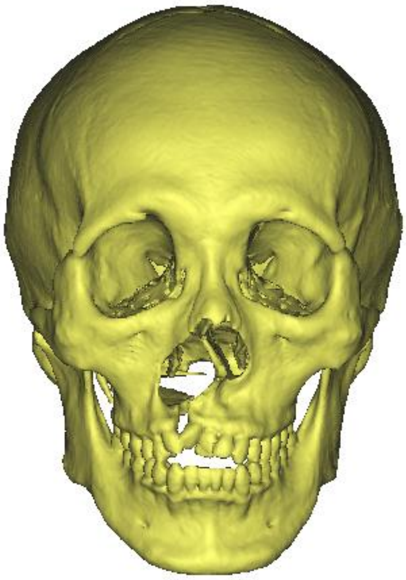	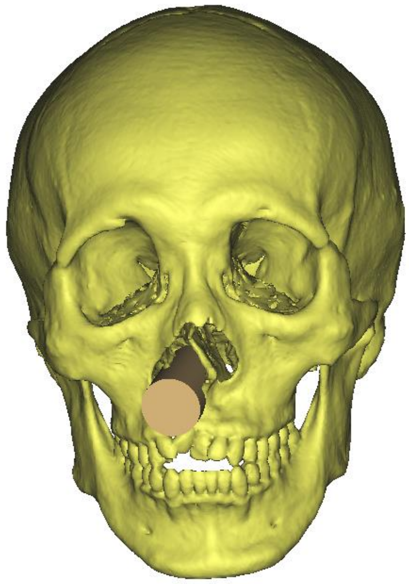

#11	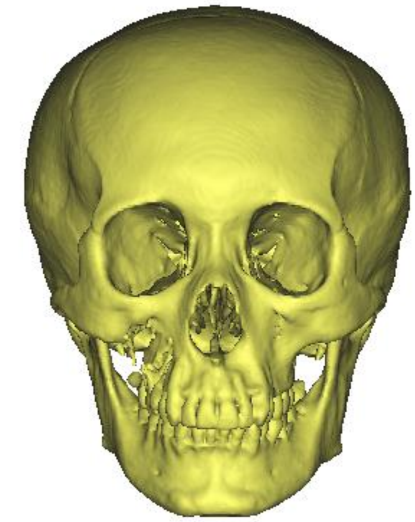	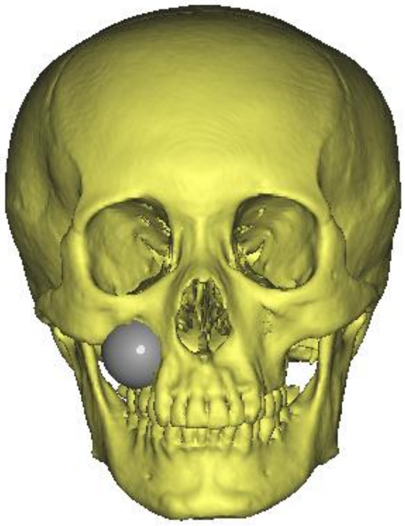
#12	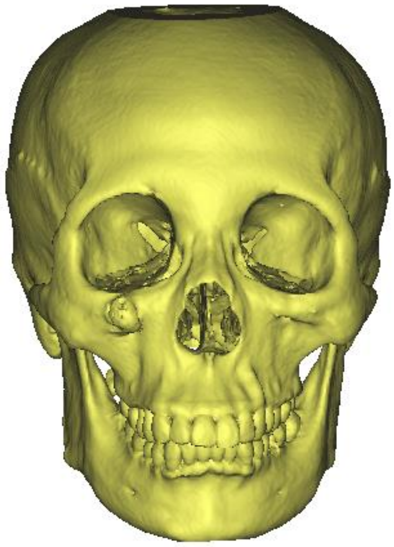	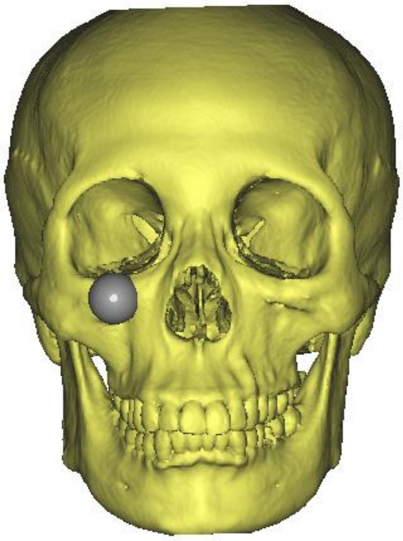

#13	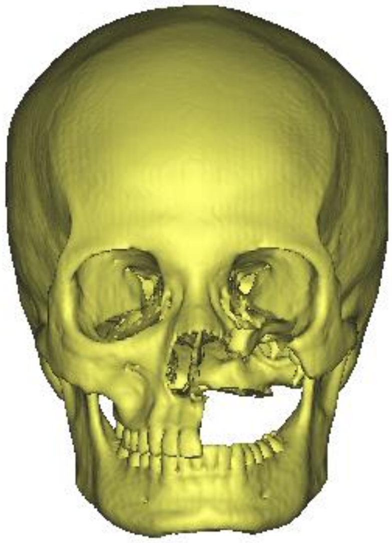	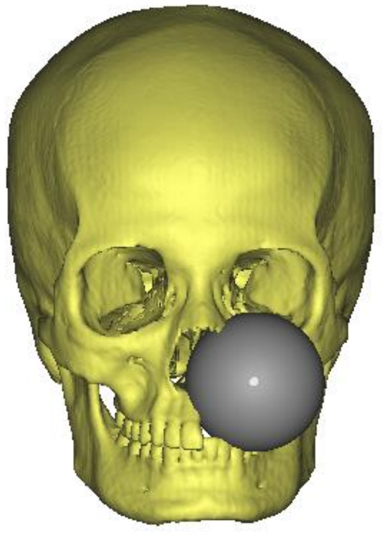
#14	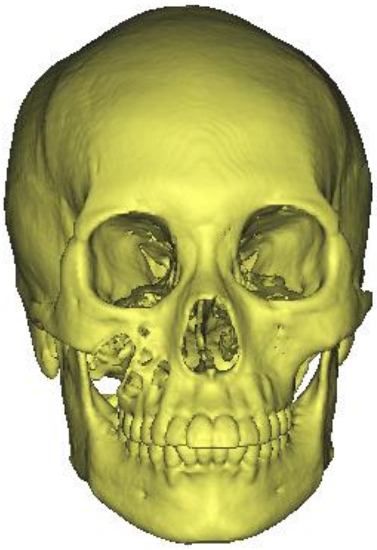	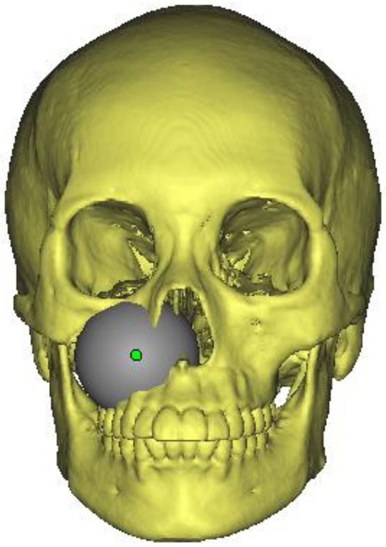

#15	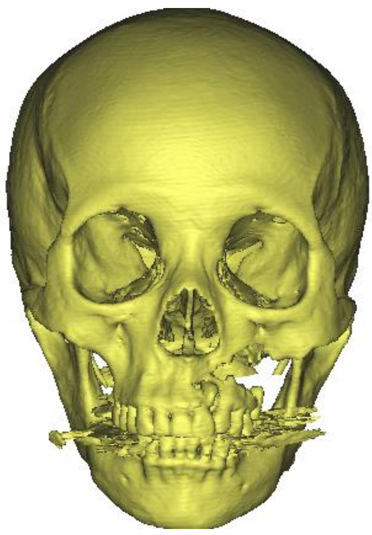	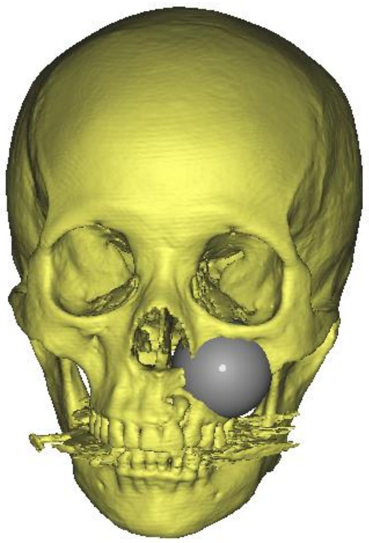
